# Results of the Scandinavian Sarcoma Group XIV protocol for classical osteosarcoma: 63 patients with a minimum follow-up of 4 years

**DOI:** 10.3109/17453674.2011.566141

**Published:** 2011-04-05

**Authors:** Sigbjørn Smeland, Øyvind S Bruland, Lars Hjorth, Otte Brosjö, Bodil Bjerkehagen, Gustaf Österlundh, Åke Jakobson, Kirsten Sundby Hall, Odd R Monge, Olle Björk, Thor A Alvegaard

**Affiliations:** ^1^Division of Surgery and Cancer Medicine, Oslo University Hospital; ^2^Institute for Clinical Medicine, University of Oslo; ^3^Department of Oncology, Norwegian Radium Hospital, Oslo University Hospital, Oslo, Norway; ^4^Department of Pediatric Oncology, Lund University Hospital, Lund; ^5^Department of Orthopaedics, Karolinska Hospital, Stockholm, Sweden; ^6^Department of Pathology, Norwegian Radium Hospital, Oslo University Hospital, Oslo, Norway; ^7^Department of Pediatric Hematology and Oncology, the Queen Silvia Children's Hospital, Sahlgrenska University Hospital, Gothenburg; ^8^Pediatric Oncology Unit, Astrid Lindgren Children's Hospital, Stockholm, Sweden; ^9^Department of Oncology, Haukeland University Hospital, Bergen, Norway; ^10^Department of Cancer Epidemiology, Lund University Hospital, Lund, Sweden

## Abstract

**Background and purpose:**

The Scandinavian Sarcoma Group (SSG) XIV protocol is based on experience from previous SSG trials and other osteosarcoma intergroup trials, and has been considered the best standard of care for patients with extremity localized, non-metastatic osteosarcoma. We analyzed the outcome in 63 consecutive patients.

**Patients and methods:**

From 2001 through 2005, 63 patients recruited from centers in Sweden, Norway, and Finland were included. They received preoperative chemotherapy consisting of 2 cycles of paired methotrexate (12 g/m^2^), cisplatin (90 mg/m^2^), and doxorubicin (75 mg/m^2^). 3 cycles were administered postoperatively, and poor histological responders were given 3 additional cycles of ifosfamide (10–12 g/m^2^) as a salvage strategy.

**Results:**

With a median follow-up of 77 months for survivors, the estimated metastasis-free and sarcoma-related survival at 5 years was 70% and 76%, respectively. 53 patients were treated with limb salvage surgery or rotationplasty and 2 patients experienced a local recurrence. 3 toxic deaths were recorded, all related to acute toxicity from chemotherapy. The 5-year metastasis-free survival of poor histological responders receiving add-on treatment with ifosfamide was 47%, as compared to 89% for good histological responders.

**Interpretation:**

Outcome from the SSG XIV protocol compares favorably with the results of previous SSG trials and other published osteosarcoma trials. However, salvage therapy given to poor responders did not improve outcome to a similar degree as for good responders. In a multi-institutional setting, more than four-fifths of the patients were operated with limb salvage surgery or rotationplasty, with few local recurrences.

Current management of high-grade osteosarcoma comprises pre- and postoperative chemotherapy and complete surgical removal of all tumor sites (Link et al. 1986, Fuchs et al. 1998, Bacci et al. 2000). With this strategy, 5-year overall survival rates of 70% have been reported for patients aged ≤ 40 years with non-metastatic, extremity localized osteosarcoma at diagnosis (Bielack et al. 2002, Smeland et al. 2003, Ferrari et al. 2005). Preoperative chemotherapy offers an opportunity to modify postoperative chemotherapy according to histological response. Although never proven to be effective in a randomized trial, this principle has been used in several osteosarcoma trials (Rosen et al. 1979, 1982, Winkler et al. 1988, Bacci et al. 1993).

The drugs active in osteosarcoma are doxorubicin, methotrexate, cisplatin, and ifosfamide, but there is still no consensus on their optimal combination (Fuchs et al. 1998, Ferrari et al. 2005, Meyers et al. 2005, Lewis et al. 2007). In the SSG VIII study, all patients received a preoperative combination of methotrexate, doxorubicin, and cisplatin (Smeland et al. 2003). Poor responders were given an exchange to a combination of ifosfamide/etoposide postoperatively. 5-year metastasis-free and sarcoma-related survival was 63% and 74%, respectively. However, the ifosfamide/etoposide replacement resulted in poor outcome for poor responders. In the recent Italian/Scandinavian ISG/SSG 1 protocol, the addition of high-dose ifosfamide up-front to all patients failed to improve outcome (Ferrari et al. 2005). In a previous trial from the Rizzoli Institute, giving ifosfamide (9 g/m^2^) to poor responders had resulted in a similar outcome for both poor and good responders (Bacci et al. 1993). This, together with the COSS-86 study using the 4 active drugs for all patients, probably represents the best survival data published (Fuchs et al. 1998). Thus, based on the SSG's own experience and that of other intergroups, the SSG XIV protocol was considered to be the best standard therapy in classical osteosarcoma. All patients received a 3-drug combination of methotrexate, doxorubicin, and cisplatin (MAP) pre- and postoperatively and poor histological responders also received high-dose ifosfamide. A primary report on the SSG XIV protocol was published in the SSG 30-year anniversary report (Smeland et al. 2009). Here we report the results with a minimum follow-up of 4 years.

## Patients and methods

### Patients

**Figure 1.**
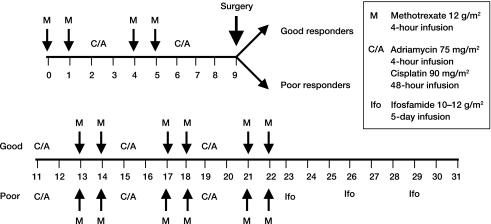
Outline of treatment in the SSG XIV protocol.

From March 2001 through April 2005, 71 consecutive patients with high-grade extremity-localized osteosarcoma from 6 centers in Sweden, 3 centers in Norway, and 2 centers in Finland, were treated according to the SSG XIV protocol (Figure 1). Eligibility criteria were age ≤ 40 years and no metastases evident at presentation, by mandatory use of chest CT and whole-body bone scintigraphy. The primary tumor was assessed by conventional radiographs, technetium 99-MDP bone scan, MRI scan of the entire bone involved, and additional CT scan when needed. The diagnosis of osteosarcoma was confirmed by open biopsy. The SSG pathology panel reviewed all cases and agreed on diagnosis, subtype, and malignancy grade. 8 patients were excluded due to metastatic disease (n = 6), a revised diagnosis of clear cell sarcoma (n = 1), or malignant fibrous histiocytoma (n = 1), thus making 63 patients (40 males) eligible for this analysis (Table). The median age at diagnosis was 15 (8–39). Primary tumor localizations were femur (n = 34), tibia (n = 15), humerus (n = 6), fibula (n = 4), radius (n = 2), and other (n = 2). The tumor volume measurements were based upon MRI scans using the ellipsoid formula V = 0.52 × abc.

**Table:** Patient characteristics

	No.
Patients	63
Sex	
Male	40
Female	23
Median age (range)	15 (8–39)
Site	
Femur	34
Tibia	15
Humerus	6
Other	8
Histology	
Osteoblastic	48
Chondroblastic	4
Fibroblastic	2
Telangiectatic	1
Other/combinations	8
Median tumor volume, mL (range)	130 (8–858)

### Chemotherapy

Chemotherapy consisted of 2 cycles of methotrexate (MTX; 12 g/m^2^), doxorubicin (ADM; 75 mg/m^2^), and cisplatin (CDP; 90 mg/m^2^) (MAP) preoperatively and 3 cycles postoperatively (Figure 1). Poor histological responders continued to receive 3 courses of ifosfamide (IFO; 10 g/m^2^) as an add-on treatment.

With good hematological tolerance, the protocol advocated escalation of the IFO dose by 20% for the next course. MTX was administered in a 4-hour infusion with 11 doses of leucovorin (folinic acid) as rescue (8 mg/m^2^) every sixth hour, beginning 24 h after starting the MTX infusion (Ferrari et al. 2005). CDP was delivered as a 48-h continuous infusion intravenously and was followed by ADM given as a 4-hour continuous infusion. IFO, in combination with an equal amount of mesna, was administered as continuous infusion at a dose of 2 g/m^2^/day for 5 consecutive days. Postoperative chemotherapy was scheduled to begin 1 week after surgery. All drugs were given as single agents.

Complete blood counts, renal function, and liver function were monitored before each chemotherapy cycle. No dose reduction was allowed, and if the absolute granulocyte count was equal to or less than 1000/μL (500 for MTX cycles), and/or the platelet count was equal to or less than 100.000/μL (60.000 for MTX cycles), chemotherapy was delayed until recovery. After each cycle, the blood count was monitored twice weekly starting on day 7 from the start of chemotherapy. G-CSF support was given according to the ASCO guidelines (1994).

### Surgery and histological response assessment

The type of surgery was chosen depending on the size and the location of the tumor, neurovascular involvement, and skeletal maturity. For local excision, it was mandatory that preoperative staging gave the possibility of achieving adequate surgical margins. The type of reconstruction was chosen according to tumor location and extension, patient age, and preferences. After surgery, the surgical margins were assessed according to Enneking as radical, wide, marginal, or intralesional. The grading of histological response analysis was done with a modified 2-grade Huvos scale (Huvos et al. 1977). Good response was defined as < 10% of examined tumor area revealing unquestionable viable tumor and no single area of unaffected viable tumor exceeding 2.5 mm in its largest diameter. Poor response was defined as either of the 2 criteria being unfulfilled. The initial pathological evaluation at each institution determined the postoperative chemotherapy. Sections from each patient's tumor were reviewed for histological response by the SSG reference pathology panel.

### Response criteria and statistical analysis

Metastasis-free survival was calculated from the date of diagnosis of the primary tumor until the date of diagnosis of distant metastases or last follow-up. Event-free survival was calculated from the date of diagnosis until the date of distant metastases, local recurrence, treatment-related death, or last follow-up. Sarcoma-related survival was calculated from the date of diagnosis until death from osteosarcoma, from treatment-related causes, or last follow-up. For statistical analysis, SPSS for Windows release 15.0 was used. The Kaplan-Meier method was used for survival analysis and 95% confidence intervals (CIs) were calculated. Survival distributions were compared by the log-rank test.

## Results

Clinicopathological data are listed in the Table.

### Compliance

2 patients did not receive neoadjuvant chemotherapy, as one was in need of prompt surgical treatment and the other had a preoperative diagnosis of chondrosarcoma, which was later revised to high-grade osteosarcoma. 1 patient was treated postoperatively according to a different chemotherapy protocol, as decided by the treating physician. The median time from start of chemotherapy to surgery was 80 days, which represents a median delay of 17 days to protocol. The median duration of treatment was 219 days in good responders and 275 days in poor responders, representing median delays of 65 days for good responders and 67 days for poor responders. Data on chemotherapy compliance were available for 57 patients. Of these, 50 patients received all 5 courses of CDP/ADM, of whom 37 were given with no dose reduction. 2 patients received only 2 courses, one due to early death and the other due to change in postoperative therapy as mentioned above. 5 patients received only 4 courses due to toxicity. 47 of the 63 patients received all 10 courses of MTX. No dose reduction was recorded for MTX. Regarding IFO courses, 23 of the 26 patients received all 3 courses with a dose reduction for 3 of the patients. Overall, 49 of the 57 patients included in the compliance analyses received 4 or more courses of CDP/ADM and 8 or more courses of MTX.

### Toxicity

Detailed toxicity data were available for 48 of the patients, and included 233 ADM/CDP courses in total. One third of the CDP/ADM courses and one quarter of the IFO courses were followed by grade-IV leukopenia. 10 of the patients did not experience any episode of grade-IV leukopenia. Regarding platelet toxicity, 28% of the CDP/ADM courses were followed by grade-IV thrombocytopenia and 31% of patients received platelet transfusion. For the IFO courses, only 1 out of 81 recorded courses was followed by grade-IV toxicity and no platelet transfusion was given. 9 patients experienced a mild to moderate renal impairment, mainly after MTX administration. This caused changes in the planned schedule for 1 patient. None of these patients required dialysis.

We experienced 3 treatment-related deaths, all related to acute toxicity from chemotherapy. 1 patient (a boy of 8 years) developed neutropenic fever and septic shock after completion of preoperative therapy. 1 patient (a good responder) developed typhlitis and septicemia after the first postoperative cycle of chemotherapy, and 1 patient (a poor responder) developed septic shock in association with the last cycle of postoperative ADM/CDP. In addition, 2 cases of life-threatening toxicity were observed. A male patient (aged 21) stayed 10 days at the intensive care unit after completion of preoperative chemotherapy due to development of severe enterocolitis in combination with grade-IV neutropenia and thrombocytopenia. The second case was a girl (aged 15) who developed grade-IV cardiotoxicity before the third course of IFO, and who was in need of prompt medical treatment. Her cardiac function is not fully normalized more than 3 years after the end of therapy, and the patient is permanently in need of medical treatment.

### Surgery and local control

53 of the patients were treated with limb salvage surgery (n = 45) or rotationplasty (n = 8). 8 patients had amputations and 2 patients were not operated due to early progression or toxic death. 39 of the operated patients obtained wide margins. 2 patients, both poor responders, developed local recurrence at 18 and 29 months after diagnosis. One had a wide surgical margin and the other had a marginal one. 1 of these patients later developed distant metastases and died 30 months after diagnosis. The other was treated with amputation (lower extremity), and is alive in second complete remission 64 months from diagnosis and 35 months from local recurrence. The 5-year projected local recurrence-free survival is 96% (CI: 91–100).

### Histological response and postoperative chemotherapy

Histological response was documented in 59 patients. As mentioned above, 2 patients were treated with amputation. 1 patient had toxic death before surgery and one patient was never operated due to early disease progression. According to the primary response assessment that decided the postoperative therapy, 29 patients achieved a good response and 30 patients had a poor response. Information on postoperative chemotherapy was lost for 1 patient (a poor responder). For the remaining 58, in 1 poor responder alpha-interferon was exchanged for IFO as salvage therapy, 1 poor responder received postoperative chemotherapy according to another protocol, and 1 poor responder died before the start of IFO. Thus, 29 patients received unchanged MAP postoperatively and 26 patients underwent salvage therapy with addition of IFO according to protocol. In 5 of 26 patients, the IFO dose was escalated by 20% for the second and/or third cycle. No patient had a second escalation of the IFO dose. The pathology review process changed the response assessment in 4 patients, in 3 from good to poor response and in 1 from poor to good response. Thus, according to definite response assessment by review pathologists, 27 of the patients had a good response and 32 had a poor response.

### Survival and post-relapse outcome

**Figure 2.**
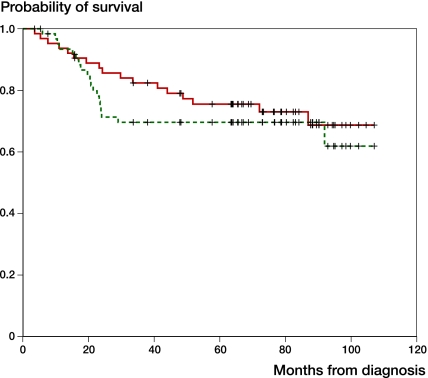
Sarcoma-related (red) and metastasis-free (green) survival (n = 63).

With a median follow-up time of 77 (16–107) months for survivors, 45 patients are alive and in complete remission. Excluding 1 patient who was lost to follow-up at 16 months, the follow-up time for survivors was at least 4 years. 1 patient died, not related to osteosarcoma or actual treatment. The estimated sarcoma-related survival at 5 years is 76% (CI: 65–87) (Figure 1). 19 patients experienced a metastatic relapse and the projected metastasis-free survival at 5 years is 70% (CI: 58–82) (Figure 2). The median time from diagnosis to relapse was 20 months, and 18 relapses occurred within 30 months from diagnosis—and in 1 patient, 92 months from diagnosis. The event-free survival at 5 years was 65% (CI: 53–77), including 1 local recurrence and 3 treatment-related deaths in addition to 19 metastatic relapses. 5-year metastasis-free survival by postoperative chemotherapy (n = 55) was 89% (n = 29) for patients receiving unchanged MAP and 48% (n = 26) for patients receiving addition of IFO (log rank test, p = 0.003). Five-year metastasis-free survival by definite histological response (n = 59) was 89% (n = 27) for good responders and 54% (n = 32) for poor responders (log rank test, p = 0.01). For the definitely poor responders who received salvage therapy with IFO (n = 24), the 5-year metastases-free survival was 48%.

Metastasis-free survival revealed no differences between sexes, with 5-year survival of 68% for males and 73% for females (log rank test, p = 0.7). Of the 19 patients who developed distant metastases, 13 had lung metastases only, 3 had lung metastases in combination with metastases at other sites, 2 had bone metastases only, and no information was available for 1 patient. Treatment of relapse was not defined by protocol and it varied between centers according to previous therapy; 15 patients were treated using second-line surgery and surgery only was offered to 7 patients. Second-line chemotherapy was given to 7 patients and 1 patient received interferon adjuvant to surgery. 14 patients achieved a second complete remission and 6 of those are alive 16–74 months after relapse. All 5 patients who did not have a second complete remission died of osteosarcoma.

## Discussion

The characteristics of the patients in the present SSG XIV study are similar to those in the SSG VIII and ISG/SSG 1 studies, and the survival data are similar to those in other osteosarcoma trials (Saeter et al. 1991, Fuchs et al. 1998, Smeland et al. 2003, Ferrari et al. 2005, Meyers et al. 2005). The SSG XIV 5-year sarcoma-related survival was 76% and metastasis-free survival was 70%, as compared to 64% and 55% in the SSG II study (Saeter et al. 1991), 74% and 63% in the SSG VIII study (Smeland et al. 2003), and 72% and 60% in the ISG/SSG 1 study (Ferrari et al. 2005).

Metastasis-free survival probably best reflects the efficacy of the chemotherapy regimen and, interestingly, the metastasis-free survival was higher in SSG XIV using a 3-drug regimen for all patients, with addition of IFO only for poor responders, as compared to the ISG/SSG 1 trial that used all 4 drugs up-front. This may reflect the possibility that not only cumulative doses but also the dose intensity of each drug is important (Bruland and Pihl 1997).

In previous trials, the amputation rates were much higher: e.g. the SSG II study had an amputation rate of 77% and a local recurrence rate of 5%. The fraction of SSG XIV patients that received limb salvage surgery or rotationplasty (0.8) was comparable to that in the ISG/SSG 1 trial (0.9%) with a local recurrence rate of 3% and 5%, respectively. Thus, it seems appropriate to conclude that in a multi-institutional setting more than four-fifths of the patients can be offered limb-salvage surgery or rotationplasty without altering the risk of local recurrence. The low number of local recurrences in SSG XIV may also have contributed to the favorable sarcoma-related survival rate, since local recurrences are often followed by distant metastases and death from disease (Bacci et al. 2007). In SSG XIV, 2/63 patients had a local recurrence in contrast to 8/113 in the SSG VIII study.

Another issue that may influence the overall results is stage migration due to the consistent use of high-resolution CT chest scans. Patients with metastases at presentation have a worse prognosis, and for the 6 patients who were not included in this analysis because of metastatic disease at presentation (but who were treated according to the protocol), 4 died from their disease.

Treatment of metastatic relapse was not defined by the protocol, but the data reveals a substantial practice of metastasectomy. 14 of 19 relapsed patients had a second complete remission and, of these, six are alive. This is comparable with published results from other intergroups and SSG's own experience (Saeter et al. 1995, Ferrari et al. 2003, Kempf-Bielack et al. 2005). In SSG VIII, three quarters of the relapsed patients received second-line surgery and the overall survival at 5 years from relapse was 21%; all were in the group that had a second complete remission (Smeland et al. 2003).

The preoperative chemotherapy in this protocol was similar to that in SSG VIII. However, the percentage of good responders differed—46% in SSG XIV and 58% in SSG VIII. This may reflect the slightly modified pathology assessment criteria defined by this protocol.

The difference in metastasis-free survival between good and poor histological responders in the current report was 42%. For the patients with a definite assessment of poor histological response who received additional IFO treatment, 5-year survival was 48%. With the limitation of a non-randomized design, and based on a univariate analysis only, the data show that the salvage strategy used in this protocol did not improve the prognosis of poor responders to that of good responders. Interestingly, the 5-year metastases-free survival for poor responders in the SSG VIII study that were salvaged by a postoperative exchange of MAP (used preoperatively) to an ifosfamide/etoposide combination showed a similar metastases-free survival of 53% (Smeland et al. 2003). This may reflect an underlying chemoresistance, including one to IFO. The reason for the design chosen in SSG XIV was not to interfere with the dose intensity of MAP. This highlights the importance of the ongoing EURAMOS-1 study (Marina et al. 2010), which will address the salvage question in a randomized trial. Compared to the SSG XIV protocol, IFO is introduced earlier in the postoperative regimen in EURAMOS-1.

We recorded 3 treatment-related deaths in SSG XIV. The reported percentage of treatment-related death in previous SSG osteosarcoma trials was in the range of 1–3%: 3/113 in SSG VIII and 1/57 in ISG/SSG I. 2 more cases of life-threatening toxicities in the current trial, all due to acute chemotherapy-induced toxicity, emphasize the importance of organizing safe osteosarcoma care. The 4 cases with life-threatening enterocolitis combined with neutropenic fever reported in SSG XIV contrast with the SSG VIII study, which used the same drugs at the same doses but with differences in administration of CDP and ADM. In SSG VIII, CDP was given as a 4-h infusion instead of the 48-h treatment in SSG XIV, and ADM was administered as a daily 4-h infusion at a dose of 25 mg/m^2^ for 3 consecutive days rather than the one 4-h infusion at a dose of 75 mg/m^2^ used in SSG XIV. To our knowledge, there have no reports in the literature on more acute (or long-term) toxicity associated with a prolonged CDP infusion, as seen in SSG XIV. Regarding doxorubicin scheduling, a more prolonged infusion is associated with more acute mucocitis but less cardiotoxicity, and thus does not explain the increased intestinal toxicity observed in SSG XIV compared to SSG VIII (Bielack et al. 1996). One could speculate that the more frequent use of growth factor support in SSG XIV does not reduce the pattern of serious life-threatening toxicity but rather shifts it.

In conclusion, the outcome of the SSG XIV protocol compares favorably with previous SSG trials and other published osteosarcoma trials. In this non-randomized trial, the salvage strategy with the use of high-dose IFO as an additional treatment did not improve the outcome for poor responders to a level similar to that attained by good responders.
